# How Did Zero-Markup Medicines Policy Change Prescriptions in the Eyes of Patients?—A Retrospective Quasi-Experimental Analysis

**DOI:** 10.3390/ijerph191912226

**Published:** 2022-09-27

**Authors:** Hanchao Cheng, Yuou Zhang, Jing Sun, Yuanli Liu

**Affiliations:** School of Health Policy and Management, Chinese Academy of Medical Sciences & Peking Union Medical College, 5 Dongdansantiao, Dongcheng District, Beijing 100730, China

**Keywords:** zero-markup medicines policy, policy effect, outpatient, prescription, overuse, hospital

## Abstract

Background: China implemented the zero-markup medicines policy to reverse the overuse of medicine in public health institutions, by changing the distorted financing mechanism, which heavily relies on revenue generated from medicines. The zero-markup medicines policy was progressively implemented in city public hospitals from 2015 to 2017. Objective: This study is expected to generate convincing evidence with subjective measurements and contribute to a more comprehensive evaluation of the policy from both objective and subjective perspectives. Methods: This study was based on a large patient-level dataset with a quasi-experimental design. We employed the difference-in-difference (DID) method, combined with propensity score matching methods, to estimate the causal effect of the policy in reducing overprescriptions from the patient perspective. Results: The study estimated a statistically significant increased probability that the responded outpatients denied overprescription in their visiting hospitals. The mean interacted policy effect, in percentage points, of all observations were positive (logit DID model: 0.15, *z* = 10.27, SE = 0.01; PSM logit DID model: 0.15, *z* = 10.26, SE = 0.01; PSM logit DID hospital fixed-effect model: 0.12, *z* = 3.00, SE = 0.04). Discussion: The policy might reduce overprescription in public hospitals from the patient’s perspective. The patient’s attitude is one aspect of a comprehensive policy evaluation. The final concrete conclusion of the policy evaluation can only be made through a systematic review of the studies with rigorous design and with both objective and subjective measurements.

## 1. Background

China implemented a low-price policy for healthcare to ensure patient affordability during the planned economy. The profit and loss of public hospitals were all borne by the government. Public hospitals have been allowed to add 15% of the procurement price of medicines dispensed by the hospital pharmacy, to compensate for insufficient government subsidy and to maintain institutional running, since 1954 [1]. Most of the prescriptions were filled by the hospital pharmacy, as the retail pharmacy was yet developed during that time. Upon entering into the market economy in the 1980s, the price of healthcare in Chinese public hospitals remained at a low level, which was far below the real cost. However, the government subsidy to healthcare kept decreasing. Public hospitals were allowed to have the rebate from pharmaceutical suppliers accounted for up to 5% of the transaction cost [2]. To motivate the public hospitals and to improve the efficiency of healthcare service, the government allowed public hospitals to keep the surplus from the markup and rebate of medicines to reward staff. Public hospitals, therefore, heavily relied on the revenue generated from medicines, which motivated overprescription (unnecessary medication, especially those high-priced medications) and led to potential negative implications of quality of care [3].

The government introduced a series of healthcare reforms in 2009 to change the distorted financing mechanism, which heavily relies on revenue generated from medicines in public hospitals, aiming to reverses the overuse of medicines. One of the core policies was the zero-markup medicines policy (hereinafter referred to as “the policy”) [4]. The policy refers to public health facilities dispense medicines (except traditional Chinese herbal medicines) at the procurement price, and no markup is added to the medicines dispensed. Considering that public hospital pharmacies still played a key role in filling prescriptions, this policy may generally affect the revenue generation of public hospitals. So, the policy was designed to be accompanied by the increased price of healthcare service and increased government subsidy to compensate the potential loss from removing the markup of medicines [5]. The policy has been progressively implemented in the Chinese public health facilities, from the primary level to the secondary and the tertiary levels step by step. In 2009, it was part of the national essential medicines policy implemented at the primary level [6]. It was expanded to county hospitals from 2012 to 2014 [7,8] and later to city hospitals [9]. All public hospitals implemented the policy by the end of September 2017 [10].

Many studies evaluated the impact of the policy on the service utilization, level of expenditures, and economic performance of public hospitals [11,12,13]. Apart from objective measurements, there was a limited number of studies that adopted subjective measurements, which surveyed improved patient satisfaction and doctor satisfaction after implementing the policy [14,15,16,17]. However, none of these analyses of subjective measurements were with an appropriate control design to reduce the potential confounders. This study generated subjective evidence based on a large patient level dataset with a quasi-experimental design. The change of patient’s perspective towards overprescription in public hospitals after implementation of the policy will contribute to a more comprehensive and solid evaluation, if the policy objective of reversing overprescription is achieved from both the objective and subjective perspectives.

## 2. Methods

### 2.1. Study Design

The study assumed that implementation of the policy might change the financing model of public hospital and reduce overmedication, and the changes of prescriptions might be perceived by the patient. The observation unit of this study was individual outpatient, surveyed by the same evaluation team with the same convenient sampling method and with the same survey tools and strategy, during the same months of respective year, which were regarded as independent surveys. By pooling the independent cross-sections, the study employed the two-stage difference-in-difference (DID) method [18,19] to estimate the before and after change of patient’s perception of overprescription of the visiting hospital, which started to implement the policy in 2016, by controlling the comparable outpatient in the comparable hospital that had yet to start to implement the policy in 2017. Considering that there were statistically significant differences in the distributions of the observed characteristics of the responded outpatients and their visiting hospitals between the treatment group and the control group in both 2015 and 2017, and, provided that rich data on the treatment group and the control group existed, the study combined propensity score matching (PSM) with DID methods. During the observation time of this study, except the zero-markup medicines policy, there were no other major policy changes intending to change prescription behavior.

### 2.2. Data Source

This study was based on the annual external evaluation of the National Healthcare Improvement Initiative in a cohort of 136 public tertiary hospitals across 31 provinces of China at the end of each year from 2015 to 2018. Surveys in each province were led by a local expert and another 2–5 expert members. These provincial experts were trained by the survey designer and organizer at central level. About 50 medical students were recruited as investigators in each province. Provincial experts provided training of the local investigators with unified training materials. Each year, there were about 1500 investigators carrying out the on-site investigation across the country [20]. One general hospital, one traditional Chinese medicine hospital, one maternal and child hospital of each province, and all centrally affiliated hospitals were included in the cohort. On-site outpatient surveys were performed around the hospital pharmacy window by well-trained investigators, encountering patients who completed consultations in hospitals. Supposed that 85% of outpatients gave a positive response of satisfaction towards the services, the significance level was set at 0.05 to calculate the minimum sample size, in each hospital which was 196. The planned outpatient sample size was set as 200 in each sample hospital. At least 200 outpatients responded to the outpatient survey in each included hospital. The exact starting time of the implementation of the policy in each surveyed hospital, and the hospital characteristics were obtained from the facility survey of the evaluation. Patient’s perception towards overprescription in their visiting hospital was one question of the outpatient questionnaire. The characteristics of the responded patients were obtained from the survey dataset. The reliability of the questionnaire was tested with the Cronbach’s α, which is greater than 0.9. Detailed general information are available from the academic publications about the outpatient surveys 2015–2019 [21,22,23]. The patient investigation protocol was reviewed and approved by the Ethics Committee of Peking Union Medical College (SPH201611CHII206). The purpose and organization of the survey were stated at the beginning of the questionnaire, and all respondents were informed and consented to respond through agreement of a preset question before they initiated the survey.

### 2.3. Participants and Setting

Considering the lagged implementation of the policy, the surveyed hospitals that started to implement the policy in the first half of the year (before June 30) were regarded as having started to implement the policy in the same year, otherwise (after June 30) they were regarded as having started to implement the policy in the next year. Therefore, 13 surveyed hospitals started to implement the policy in 2015, another 17 started in 2016, 39 implemented in 2017, and all had implemented by 2018 (Table S1). The study regarded 2015 as the baseline year and 2016 as the intervention time. The study constructed the pooled cross-sectional data by assigning the 17 surveyed hospitals that started to implement the policy in 2016 as the treatment group, and the 39 that had started to implement in 2017 as the control group. As there were no centrally affiliated specialty hospitals other than traditional Chinese medicine (TCM) or maternal and child health (MCH) in the treatment group, the study excluded three of such hospitals from the control group (Table S2), thus, the study assigned 17 and 36 surveyed hospitals to the treatment group and the control group, respectively. The sample patients were, therefore, broken down into four groups: patients in the treatment group and the control group, before and after 2016. The study extracted the 2015 and 2017 outpatient survey results about patient’s perceptions towards overprescription in the visiting hospital and estimated the change in the patient’s perception in the surveyed hospitals, which implemented the policy for at least one and a half years. The characteristics of the responded outpatients in the treatment group (10,617) and the control group (11,154) in 2015 and 2017 were presented in Table S3. The sample construction and data extraction strategies were based on several considerations. One was that the surveyed hospitals started to implement the policy progressively, and all had implemented the policy by the end of September 2017. Although 13 surveyed hospitals started to implement the policy in 2015, the baseline data in 2014 were not available, and there were no hospitals in non-implemented status that could be set as a control in 2018. So, the possible intervention time would have to be 2016 or 2017. Secondly, the policy effect might be lagged, and the immediate effect in the surveyed hospitals that started to implement the policy in 2017 might not be fully shown. So, the study chose 2016 but not 2017 as the intervention time for estimation. Thirdly, the national mainstream media (China Central Television, CCTV) reported adverse news about the unethical and illegal kickbacks associated with high prices of medicines halfway through the 2016 survey. The CCTV report negatively affected the patient’s perception of the patient–doctor relationship [24] and might very likely negatively affect the patient’s perception toward overprescription in the 2016 survey. So, the study did not extract the 2016 outpatient survey results and did not estimate the short-time policy effect in the surveyed hospitals that started to implement the policy in 2017.

### 2.4. Measurement

The outcome measurement of the logit DID analysis was a dichotomous variable. The surveyed outpatients were asked whether or not they agreed that there were overprescriptions in their visiting hospitals. The study defined disagreement as 1 and agreement as 0. The explanatory variables included two conditional variables, the time variable and the policy variable. The study defined the time variable as 1 for the year 2017 and 0 for the year 2015. The study defined the policy variable as 1 for the treatment group and 0 for the control group.

To perform the PSM, the study defined the treatment group as 1 and the control group as 0. The study had the demographic and socioeconomic characteristics of the responded outpatients and the characteristics of their visiting hospitals as covariates to fit the logistic regression model and calculated the likelihood of the responded outpatients who disagreed that there were overprescriptions in their visiting hospitals.

### 2.5. Statistical Analysis

The study firstly fitted a multivariate logit model as the baseline model with the two-period cross-sectional data pooled over 2015 and 2017. The study performed the pooled logit regression by controlling all the observed characteristics of the responded outpatients and their visiting hospitals as covariates with the following equation:

(1) $$\mathrm{Logit}\left(\pi \right)=\beta_{0}+\delta_{0}time+\beta_{1}policy+X_{patient}+X_{hospital}+e$$

where π represents the probability of the responded outpatients who disagreed that there were overprescriptions in their visiting hospitals. *Time* indicates the year 2015 (before the treatment group started to implement the policy in 2016, defined as 0) and 2017 (after the treatment group started to implement the policy in 2016, defined as 1). *Policy* reflects treatment group (defined as 1) and control group (defined as 0). $X_{patient}$ and $X_{hospital}$ denote the controlled variables at the patient and the hospital level, respectively. *e* is the random error term.

The study then added an interaction term of the time variable and the policy variable into model 1 to fit the logit DID model (model 2) with the following equation:

(2) $$\mathrm{Logit}\left(\pi \right)=\beta_{0}+\delta_{0}time+\beta_{1}policy+\delta_{1}time\times policy+X_{patient}+X_{hospital}+e$$

where time*policy is the interaction term. *δ*_1_ on the interaction term quantifies the effect of the policy. $X_{patient}$ and $X_{hospital}$ denote the controlled variables at the patient and the hospital levels, respectively. *e* is the random error term [25,26,27].

As presented in Table S3, the chi-squared tests proved statistically significantly different distributions of most of the characteristics of the responded outpatients and their visiting hospitals between the treatment group and the control group in both 2015 and 2017. This implied that the parallel-trend assumption of applying the DID method might not hold. The study performed the PSM before running the DID regression (model 3) to secure the comparability of the observations between the treatment group and the control group in both 2015 and 2017. The study constructed a set of balanced observations in the treatment group and the control group, with a series of observed characteristics of the responded outpatients and their visiting hospitals as covariates in the PSM model. The combination of PSM with DID methods kept better comparability of the responded outpatients in the treatment group and the control group and resolved the non-random assignment problem that might bias the policy effect [28,29,30]. The study explored the 1:1 nearest-neighbor matching method within multiple calipers (0.10 σ, 0.05 σ, 0.01 σ) and the Kernel-matching method [31] to perform the matching, finally identifying the Kernel matching (epan, bandwidth = 0.06) that achieved the best balance with the paired *t*-test. Table S4 presented the PSM results and the balance test results. There was no statistically significant difference in each subgroup of the observed demographic and socioeconomic characteristics of the matched outpatients and the characteristics of their visiting hospitals between the treatment group and the control group.

Since only limited characteristics of the surveyed hospitals were available, to reduce the potential bias associated with the unobservable time-invariant variables of the surveyed hospitals, the study employed the hospital dummy variable method [18,19,32,33] to fit the hospital fixed-effect logit DID model, as expressed in the following equation (model 4):

(3) $$\mathrm{Logit}\left(\pi \right)=\beta_{0}+\delta_{0}time+\beta_{1}policy+\delta_{1}time\times policy+X_{patient}+D_{hospital}+e$$

$D_{hospital}$ are the dummy variables of the 17 surveyed hospitals assigned to the treatment group, and the 36 surveyed hospitals assigned to the control group. *e* is the random error term.

The study set a statistically significant level at *α* = 0.05 and used STATA15 to perform the statistical analyses.

Since the policy effect estimated by the non-linear model was not constant but was conditional on the independent variables, the coefficient of the interaction term in the logit DID model was not representative of the real magnitude of the policy effect. The statistical significance of the interaction effect of the logit model also could not be tested with a *t*-test on the coefficient of the interaction term *δ*_1_. Instead, the study run the command of “inteff” after fitting the logit DID model with the same variable list [34,35,36,37]. This command computed the accurate magnitude and statistical significance of the policy effect with the interaction effect of policy and time for each observation and also enabled for plotting of the distribution of the interaction effect and the *z*-statistic of each observation against the predicted probability that the dependent variable = 1 for models 2–5, respectively. *z* > 1.96 was implied as statistically significant.

## 3. Results

### 3.1. Responded Outpatients Included in the Study before and after Performing the PSM

The study included 10,617 responded outpatients surveyed in 2015 and 11,154 outpatients surveyed in 2017. Before performing the PSM, 3402 and 3594 responded outpatients were assigned to the treatment group, and 7215 and 7560 responded outpatients were assigned to the control group in 2015 and 2017, respectively (Table S3). After performing the PSM, 126 and 75 responded outpatients were off-support in 2015 and 2017. As a result, 7089 and 7489 responded outpatients in the control group were matched with the 3402 and 3590 responded outpatients in the treatment group in 2015 and 2017, respectively. After performing the PSM, the percent bias of most covariates was below 5%.

### 3.2. Regression Results

As presented in Table 1, before performing the PSM, the baseline logit regression (Model 1) pooled the 2015 and 2017 data and controlled the time variable and the demographic and socioeconomic characteristics of the responded outpatients as well as the characteristics of their visiting hospitals. The coefficient of the policy variable (β_1_ = 0.22, *p* < 0.01) of model 1 and the coefficients of policy and time interaction term of models 2–5 (*δ*_1_ ranged between 1.30 to 1.35, all *p* < 0.01) were all statistically significantly positive. The results of all models implied a positive policy effect, while the baseline pooled regression overestimated the policy effect. 

Considering that the coefficient of the interaction term of the nonlinear logit DID model is not appropriate to quantify the policy effect directly, the study reported the mean, the minimum, and the maximum policy effects of all observations and calculated the *z*-statistics, both of which varied largely across the observations (Table 2 and Figure S1). The mean policy effects in percentage points of all the observations calculated based on models 2–4 were all statistically significantly positive (logit DID model: 0.15, *z* = 10.27, SE = 0.01, no. of observations = 21,770; PSM logit DID model: 0.15, *z* = 10.26, SE = 0.01, no. of observations = 21,570; PSM logit DID hospital fixed-effect model: 0.12, *z* = 3.00, SE = 0.04, no. of observations = 21,770. All *p* < 0.05).

## 4. Discussion

The finding of this study verified our hypothesis that the policy of revoking the markup and rebate of medicines reduced the overuse of medicines in public hospitals. Our finding is consistent with the results of our former systematic review of the effect of the policy [11]. The review included quasi-experimental studies with objective measurements and found that the policy had causal effects of reduced expenditures [38,39,40,41,42,43,44,45,46,47,48] and reduced proportionate medicines expenditures of both outpatients and inpatients [38,39,40,41,49,50,51]. Studies with objective measurements also observed reduced revenue [43,52] and reduced proportionate revenue generated from medicines [44,52] in public hospitals. Reduced overuse of medicines is consistent with reduced proportionate medicines expenditure and medicines revenue. Considering that, completely reversing overuse of medicines in public hospitals would need a sustaining financing mechanism. If the distorted financing mechanism has been replaced with an appropriate one, further in-depth studies would be needed. 

Some simple before and after subjective measurements found improved patient satisfaction in Beijing public tertiary hospitals [14,15] but fallen satisfaction of medical staff in county public hospitals [17]. These implied that the reduced medication and medicines expenditure in public hospitals might be welcomed by the patients. However, the reduced revenue of public hospitals might discourage the healthcare staff. Appropriate pricing of healthcare services and government subsidies might not be in place to compensate the revenue lost from the implementation of the policy. Considering that pricing reform of healthcare services and government subsidies are the essential backups of the sustainability of the policy and the indispensable conditions to shift the financing model from three sources of funding to two sources of funding, whether or not the policy objective would need to be verified with further studies.

The strength of this study is that the study employed several models to estimate the policy effect and analyzed how different models helped to minimize the potential bias. The baseline model simply pooled the 2015 and 2017 survey data to run the logit regression model, which accounted for neither the unobserved time-invariant hospital-level characteristics nor the partial effect of the dependent variable concerning the time variable depending on the magnitude of the policy variable [18,19]. By employing the DID model, the study replaced the policy variable with an interaction term, which allowed the partial effect of the time variable to depend on the level of the policy variable, thus controlling for the systematic differences of the observations between the treatment group and the control group and evaluating the two differences simultaneously [26,27]. The PSM method secured that patients in the control group and treatment group were balanced enough to minimize the potential selection bias. The hospital dummy variable model captured the unobserved time-invariant characteristics of hospitals [18,19].

This study also examined the interacted policy effects graphically as an informative supplement. The graphs intuitively showed the difference of the correct estimation with the “inteff” command and the incorrect estimation with the conventional method for the linear model. The graphs also intuitively showed the distributions of the *z*-statistics of all observations. Models 2–4 all estimated a statistically significantly positive mean policy effect that increased the probability of the responded outpatients to disagree that there were overprescriptions in their visiting hospitals. The conventional marginal effect estimation method overestimated the policy effects of most observations and underestimated the policy effects of some observations that had predicted probabilities over 0.8 when the dependent variable = 1. None of the observation had a negative policy effect.

This study had several limitations. First, it was based on a survey that was designed not specifically for the causal effect analysis of the policy. The survey only covered public tertiary hospitals in large cities and did not include lower levels of care and public hospitals in rural areas. The circumstances of public hospitals in rural areas and at lower levels might not be the same case as those of public tertiary hospitals in large cities [53]. Although the first group of 13 surveyed hospitals started to implement the policy in 2015, the study could not estimate a long-term policy effect on these 13 hospitals, because baseline data in 2014 were not available. Besides, the study excluded the data for 2016. Thus, the multiple cross-sectional designs that would have been used to remove the bias from the potential time-varying confounders of the estimation was not possible. The study design was shifted from a staggered DID analysis, based on a four-year cross-sectional dataset from 2015 to 2018, into a two stage before-and-after DID analysis, based on the two-year cross-sectional dataset of 2015 and 2017 [23,26,54].

Secondly, the common trend assumption of the DID method requires the potential confounders varying across the treatment and control groups be time-invariant, and the potential time-varying confounder to be invariant across the treatment and control groups [25]. Thus, the two-group two-year data would need to be collected from two independent cross-sectional surveys, which should randomly sample outpatients from each of the surveyed hospitals. However, this was not the case in reality. The outpatient surveys adopted the convenient sampling method to target the surveyed outpatients due to the feasibility consideration and might yield unbalanced, repeated cross-sections. Considering that the surveys were conducted by the same team with the same convenient sampling method, the same survey tools, and the same strategy, during the same months of the respective years, the study assumed that the outpatient samplings would be approximately the same as the independent draws. To improve the balance of the surveyed outpatients between the treatment and the control groups, the study adopted the PSM method to match them. However, PSM could only balance the outpatients with the available characteristic variables from the survey. Many factors that might affect the patient’s choice of hospital, such as the equipment, the reputation of the doctors, former hospital experiences, etc. were not available, and the potential bias brought by the unobserved variable could not be resolved with the PSM method. These unobserved variables might affect the attitudes of the responded outpatients towards overprescription of their visiting doctors [18,19]. However, considering that there was a very small difference between the regression results before and after PSM, which implied that matching seemed to not change the estimation very much, neglect of the potential unobserved variables of patients is acceptable.

Thirdly, the subjective attitudes of the outpatients might not be time-invariant due to many factors in the real world, which were likely affected by the social environment, and might make the key common trend assumption of DID method not hold [51]. Considering that the survey was conducted in a cohort of hospitals by the same evaluation team with the same convenient sampling method, the same survey tools, and the same strategy, during the same months of the respective year, and that the patients were matched before performing the DID regression, the study assumed that the above affections on patients could be neglected.

In addition, the patient might not be able to make a scientific judgment of prescription behavior. The patient’s attitude could be only one of the many aspects of a comprehensive evaluation of the policy, which would need further causal effect evidence, generated by studies with robust design from the perspective of doctors, as well as a systematic review of the studies with rigorous designs and with both objective and subjective measurements.

## 5. Conclusions

This study adopted different models to estimate the effect of the zero-markup medicines policy with a subjective measurement. The study drew the conclusion of a positive policy effect on reducing overprescriptions from the perspective of the patient. This conclusion is consistent with the findings of other studies using objective measurements. Patient’s attitude is just one aspect of a comprehensive evaluation of the policy. The final concrete conclusion of the policy effect would need a more comprehensive evaluation of the policy from both objective and subjective perspectives. 

## Figures and Tables

**Table 1:** Regression results with difference models.

Variable	Model 1 (Baseline Pooled Logit)		Model 2 (Logit DID)		Model 3 (PSM Logit DID)		Model 4 (PSM Logit DID Hospital Fixed-Effect)	
	Coef	SE	Coef	SE	Coef	SE	Coef	SE
Policy (Control group as reference)	0.22 ***	0.04	−0.36 ***	0.06	−0.36 ***	0.06	−0.15	0.16
Time (2015 as reference)	0.07 *	0.04	−0.30 ***	0.05	−0.31 ***	0.05	−0.31	0.22
Policy × Time	/	/	1.30 ***	0.09	1.30 ***	0.09	1.35 ***	0.38
Gender (Female as reference)	−0.14 ***	0.05	−0.14 ***	0.05	−1.33 ***	0.05	−0.13 ***	0.05
Age (Younger than 18 years old as reference)								
18–35 years old	−0.38 *	0.21	−0.37 *	0.21	−0.36 *	0.21	−0.38	0.27
36–50 years old	−0.27	0.21	−027.	0.21	−0.25	0.21	−0.29	0.27
51–65 years old	−0.28	0.22	−0.31	0.22	−0.29	0.22	−0.38	0.27
Older than 65 years old	−0.26	0.22	−0.30	0.22	−0.29	0.22	−0.27	0.29
Education (Postgraduate and above as reference)								
Undergraduate	0.18 **	0.09	0.20 **	0.09	0.20 **	0.09	0.32 ***	0.10
Technical school	0.18 *	0.10	0.18 *	0.10	0.18 *	0.10	0.33 **	0.13
High school	0.16	0.10	0.18 *	0.10	0.18 *	0.10	0.30 **	0.12
Junior high school	0.10	0.11	0.11	0.11	0.11	0.11	0.30 **	0.13
Primary school and below	−0.15	0.11	−0.13	0.11	−0.12	0.11	0.20	0.13
Income level ( Below USD 3000 as reference)								
USD 3000–USD 9000	0.37 ***	0.06	0.32 ***	0.06	0.32 ***	0.06	0.24 ***	0.07
USD 9000–USD 18,000	0.45 ***	0.07	0.36 ***	0.07	0.36 ***	0.07	0.35 ***	0.10
Above USD 18,000	0.26 ***	0.07	0.17 **	0.07	0.17 **	0.08	0.24 *	0.12
Insurance coverage (Free medical care as reference)								
Formal employee program	−0.04	0.07	−0.04	0.07	−0.04	0.07	−0.02	0.09
Resident program	−0.00	0.07	−0.03	0.07	−0.04	0.07	−0.01	0.10
Other coverage	0.32 **	0.15	0.29 *	0.15	0.28 *	0.15	0.08	0.17
No coverage	−0.16 **	0.08	−0.21 **	0.08	−0.23 ***	0.08	−0.24 **	0.11
Department (Internal medicine as reference)								
Surgery	0.07	0.07	0.11	0.07	0.11	0.07	0.07	0.09
Obstetrics and gynecology	0.10	0.06	0.03	0.06	0.03	0.06	−0.12	0.09
Pediatric	0.10	0.08	0.14 *	0.08	0.14 *	0.08	0.07	0.13
Other departments	1.11 **	0.05	1.13 **	0.05	0.13 **	0.05	0.12 *	0.07
Type of hospital (General hospital as reference)								
MCH hospital	−0.17 ***	0.06	−0.17 ***	0.06	−0.16 ***	0.06	-	-
TCM hospital	0.28 ***	0.05	0.29 ***	0.05	0.29 ***	0.05	-	-
Affiliation of hospital (Central affiliation as referene)								
Local affiliation	0.24 ***	0.08	0.24 ***	0.08	0.24 ***	0.08	-	-
Region (Eastern as reference)								
Central	0.00	0.06	−0.00.	0.06	−0.02	0.07	-	-
Western	−0.54 ***	0.05	−0.55 ***	0.05	−0.55 ***	0.05	-	-
Constant	1.57 ***	0.25	1.82 ***	0.25	1.80 ***	0.25	2.11 ***	0.31

**Notes:** *** *p* < 0.01, ** *p* < 0.05, * *p* < 0.1; model 1 = baseline logit model; model 2 = logit DID model; model 3 = PSM logit DID model; model 4 = PSM logit DID hospital fixed-effect model; Coef = coefficient DID = difference-in-difference; PSM = propensity score matching; MCH = maternal and child health; TCM = traditional Chinese medicine.

**Table 2:** The magnitude of the interacted policy effect by running “inteff” command of STATA (percentage points).

	Number of Observations	Mean	SD	Min	Max
Model 2 (logit DID)					
Interacted policy effect	21,770	**0.15**	0.04	**0.05**	**0.30**
SE of the interacted policy effect	21,770	0.01	0.002	0.01	0.03
*z*-statistic of the interacted policy effect	21,770	10.27	1.30	3.87	13.95
Model 3 (PSM logit DID)					
Interacted policy effect	21,570	**0.15**	0.04	**0.05**	**0.30**
SE of the interacted policy effect	21,570	0.01	0.003	0.01	0.03
*z*-statistic of the interacted policy effect	21,570	10.26	1.29	3.87	13.99
Model 4 (PSM logit DID hospital fixed-effect)					
Interacted policy effect	21,570	**0.12**	0.07	**0.005**	**0.30**
SE of the interacted policy effect	21,570	0.04	0.02	0.002	0.08
*z*-statistic of the interacted policy effect	21,570	3.00	0.27	2.02	3.83

**Notes:** SD = standard deviation; Min = minimum; Max = maximum; SE = standard error; bold implies that the mean interaction effect was positive and statistically significant.

## Data Availability

Data will be available upon appropriate request from the corresponding author.

## Supplementary Materials

The following supporting information can be downloaded at: https://www.mdpi.com/article/10.3390/ijerph191912226/s1, Table S1: Surveyed hospitals implemented the policy during 2015–2018; Table S2: Surveyed hospitals assigned to the treatment group and the control group; Table S3: Summary statistics of the surveyed outpatients and their visiting hospitals in the treatment group and the control group in 2015 and 2017 before performing PSM; Table S4: Propensity score matching results and balance test results; Figure S1: Distribution of interaction effects against predicted probability and z-statistics using “inteff” command.
