# Supplementary material for: How Did Zero-Markup Medicines Policy Change Prescriptions in the Eyes of Patients?—A Retrospective Quasi-Experimental Analysis
Source: Int J Environ Res Public Health. 2022 Sep 27;19(19):12226. doi: 10.3390/ijerph191912226 (PMC9566082; doi:10.3390/ijerph191912226)
Supplement: Supplementary file 1 [file ijerph-19-12226-s001.zip › ijerph-1888231-supplementary.pdf]

How did zero markup medicines policy change prescriptions in the eyes of patients?

# - a retrospective quasi-experimental analysis

Table S1 Surveyed hospitals implemented the policy during 2015-2018

	2015	2016	2017	2018
Number of the implemented hospitals (n)	13	13+17=30	13+17+67=97	136
Number of the non-implemented hospitals (n)	123	106	39	0
Total number of the cohort of the surveyed hospitals	136	136	136	136

Table S2 Surveyed hospitals assigned to the treatment group and the control group

	Treatment group	Control group
	2 General centrally affiliated east region	3 General centrally affiliated east region
	2 General locally affiliated east region	3 General locally affiliated east region
	3 General locally affiliated west region	3 General locally affiliated central region
	2 TCM locally affiliated east region	6 General locally affiliated west region
	3 TCM locally affiliated west region	3 TCM locally affiliated east region
	2 MCH locally affiliated east region	3 TCM locally affiliated central region
	1 MCH locally affiliated central region	5 TCM locally affiliated west region
	2 MCH locally affiliated west region	3 MCH locally affiliated east region
		3 MCH locally affiliated central region
		4 MCH locally affiliated west region
**Total number of hospitals**	**17**	**36**

Notes: TCM=traditional Chinese medicine; MCH=maternal and child health;

Hospital coding: treatment/control group-series number-type of hospital-central/local affiliation-east/central/west region

Table S3 Summary statistics of the surveyed outpatients and their visiting hospitals in the treatment group and the control group in 2015 and 2017 before performing PSM

Variables	2015 (n=10 617)			2017 (n=11 154)		
	Treatment group (%)	Control group (%)	*P* value	Treatment group (%)	Control group (%)	*P* value
	n=3 402	n=7 215		n=3 594	n=7 560	
Patient's attitude towards over-prescribing of the visiting doctor			**0.00**			**0.00**
Agreement	644 (18.93)	983 (13.62)		279 (7.76)	1315 (17.39)	
Disagreement	2758 (81.07)	6232 (86.38)		3315 (92.24)	6245 (82.61)	
Gender			0.07			0.64
Female	2301(67.64)	5006 (69.38)		2293 (63.80)	4858 (64.26)	
Male	1101(32.36)	2209 (30.62)		1301 (36.20)	2702 (35.74)	
Age			0.23	0.228		**0.00**
0~17 years old	23 (0.68)	75 (1.04)		28 (0.78)	87 (1.15)	
18-35 years old	1843 (54.17)	3930 (54.47)		1826 (50.81)	4143 (54.80)	
36-50 years old	806 (23.69)	1614 (22.37)		938 (26.10)	2071 (27.39)	
51-65 years old	452 (13.29)	996 (13.80)		517 (14.39)	887 (11.73)	
Older than 65 years old	278 (8.17)	600 (8. 32)		285 (7.93)	372 (4.92)	
Education			**0.01**			**0.00**
Postgraduate & above	165 (4.85)	328 (4.55)		285 (7.93)	462 (6.11)	
Undergraduate	1598 (46.97)	3404 (47.18)		1731 (48.18)	3908 (51.69)	
Technical school	337 (9.91)	879 (12.18)		478 (13.30)	1004 (13.28)	
High school	484 (14.23)	998 (13.83)		453 (12.61)	925 (12.24)	
Junior high school	446 (13.11)	876 (12.14)		425 (11.83)	767 (10.15)	
Primary school and below	372 (10.93)	730 (10.12)		221 (6.15)	494 (6.53)	
Income level			**0.00**			**0.00**
Below $3 000	320 (9.41)	583 (8.08)		252 (7.01)	1047 (13.85)	
$3 000-$9 000	1541 (45.30)	3036 (42.08)		1683 (46.83)	3740 (49.47)	
$9 000-$18 000	814 (23.93)	2040 (28.27)		956 (26.60)	1632 (21.59)	
Above $18 000	727 (21.37)	1556 (21.57)		703 (19.56)	1141 (15.09)	
Insurance coverage			**0.00**			**0.00**
Free medical care	384 (11.29)	687 (9.52)		536 (14.91)	1070 (14.15)	
Formal employee program	1309 (38.48)	2568 (35.59)		1076 (29.94)	2166 (28.65)	
Resident program	1199 (35.24)	2413 (33.44)		1564 (43.52)	3210 (42.46)	
Other coverage	65 (1.91)	173 (2.40)		116 (3.23)	221 (2.92)	
No coverage	445 (13.08)	1374 (19.04)		302 (8.40)	893 (11.81)	
Department			**0.00**			**0.00**
Internal medicine	916 (26.93)	2369 (32.83)		1043 (29.02)	2201 (29.11)	
Surgery	338 (9.94)	676 (9.37)		311 (8.65)	900 (11.90)	
Obstetrics & gynecology	972 (28.57)	1959 (27.15)		949 (26.41)	1976 (26.14)	
Pediatric	364 (10.70)	478 (6.63)		387 (10.77)	601 (7.95)	
Other departments	812 (23.87)	1733 (24.02)		904 (25.15)	1882 (24.89)	
Type of hospital			0.21			0.22
General hospital	1401 (41.18)	3005 (41.65)		1486 (41.35)	3138 (41.51)	
MCH hospital	1000 (29.39)	2006 (27.80)		1042 (28.99)	2085 (27.58)	
TCM hospital	1001 (29.42)	2204 (30.55)		1066 (29.66)	2337 (30.91)	
Affiliation of hospital			**0.00**			**0.00**
Central affiliation	400 (11.76)	600 (8.32)		422 (11.74)	617 (8.16)	
Local affiliation	5002 (88.24)	6615 (91.68)		3172 (88.26)	6943 (91.84)	
Region			**0.00**			**0.00**
Eastern	1601 (47.06)	2402 (33.29)		1655 (46.05)	2484 (32.86)	
Central	200 (5.88)	1803 (24.99)		228 (6.34)	1883 (24.91)	

Table S4 Propensity score matching results and balance test results

Covariates		Mean propensity score in 2015 (n= 10 491)				Mean propensity score in 2017（n=11 079）			
		Treatment group n=3 402	Control group n=7 089	%bias	*P* value	Treatment group n=3 590	Control group n=7 489	%bias	*P* value
Gender (Ref. Female)									
Male	U	0.32	0.31	3.8	0.07	0.36	0.36	0.9	0.65
	M	0.32	0.31	3.1	0.20	0.36	0.35	2.0	0.39
Age (Ref. 0~17 years old)									
18-35 years old	U	0.54	0.54	-0.6	0.78	0.51	0.55	-8.0	0.00
	M	0.54	0.54	-0.1	0.98	0.51	0.52	-3.0	0.20
36-50 years old	U	0.24	0.22	3.1	0.13	0.26	0.27	-3.0	0.14
	M	0.24	0.24	0.4	0.87	0.26	0.26	0.7	0.75
51-65 years old	U	0.13	0.14	-1.5	0.47	0.14	0.12	7.9	0.00
	M	0.13	0.13	-0.0	1.00	0.14	0.14	1.4	0.56
Older than 65 years old	U	0.08	0.08	-0.5	0.80	0.08	0.05	12.3	0.00
	M	0.08	0.08	-0.4	0.87	0.08	0.07	3.0	0.24
Education (Ref. Postgraduate & above)									
Undergraduate	U	0.47	0.47	-0.4	0.84	0.48	0.52	-7.0	0.00
	M	0.47	0.47	-0.6	0.80	0.48	0.49	-0.8	0.74
Technical school	U	0.10	0.12	-7.3	0.00	0.13	0.13	0.1	0.97
	M	0.10	0.10	-0.8	0.74	0.13	0.14	-1.3	0.58
High school	U	0.14	0.14	1.1	0.58	0.13	0.12	1.1	0.58
	M	0.14	0.14	0.1	0.97	0.13	0.12	0.4	0.86
Junior high school	U	0.13	0.12	2.9	0.16	0.12	0.10	5.4	0.01
	M	0.13	0.13	1.0	0.68	0.12	0.11	1.5	0.54
Primary school and below	U	0.11	0.10	2.7	0.20	0.06	0.07	-1.6	0.44
	M	0.11	0.11	0.3	0.89	0.06	0.06	0.4	0.86
Income level (Ref. Below $3 000)									
$3 000-$9 000	U	0.45	0.42	6.5	0.00	0.47	0.49	-5.3	0.01
	M	0.45	0.44	1.2	0.63	0.47	0.48	-2.1	0.37
$9 000-$18 000	U	0.24	0.28	-9.9	0.00	0.27	0.22	11.8	0.00
	M	0.24	0.25	-1.3	0.58	0.27	0.26	1.1	0.67
Above $18 000	U	0.21	0.22	-0.5	0.82	0.20	0.15	11.8	0.00
	M	0.21	0.22	-0.3	0.90	0.20	0.19	1.8	0.46
Insurance coverage (Ref. Free medical care)									
Formal employee program	U	0.38	0.36	6.0	0.00	0.30	0.29	2.8	0.16
	M	0.38	0.38	0.5	0.84	0.30	0.30	-0.9	0.70
Resident program	U	0.35	0.33	3.8	0.07	0.44	0.42	2.1	0.30
	M	0.35	0.35	-0.1	0.98	0.44	0.43	1.1	0.63
Other coverage	U	0.02	0.02	-3.4	0.11	0.03	0.03	1.8	0.38
	M	0.02	0.02	-0.5	0.84	0.03	0.03	0.3	0.89
Not insured	U	0.13	0.19	-16.3	0.00	0.08	0.12	-11.3	0.00
	M	0.13	0.13	-0.9	0.70	0.08	0.09	-0.9	0.69
Department (Ref. Internal medicine)									
Surgery	U	0.10	0.09	1.9	0.36	0.09	0.12	-10.7	0.00
	M	0.10	0.10	-0.2	0.95	0.09	0.09	-0.2	0.92
Obstetrics & gynecology	U	0.29	0.27	3.2	0.13	0.26	0.26	0.6	0.76
	M	0.29	0.30	-3.0	0.22	0.26	0.28	-3.7	0.12
Pediatric	U	0.11	0.07	14.5	0.00	0.11	0.08	9.7	0.00
	M	0.11	0.09	6.4	0.01	0.11	0.10	1.3	0.60
Other departments	U	0.24	0.24	-0.4	0.87	0.25	0.25	0.5	0.79
	M	0.24	0.24	-0.7	0.77	0.25	0.24	1.6	0.49
Type of hospital (Ref. General hospital)									
MCH hospital	U	0.29	0.28	3.5	0.09	0.29	0.28	3.2	0.12
	M	0.29	0.30	-0.1	0.96	0.29	0.30	-2.8	0.25
TCM hospital	U	0.29	0.31	-2.5	0.24	0.30	0.31	-2.7	0.18
	M	0.29	0.29	-0.1	0.97	0.30	0.29	1.7	0.48
Affiliation of hospital (Ref. Central affiliation)									
Local affiliation	U	0.88	0.92	-11.5	0.00	0.88	0.92	-12.0	0.00
	M	0.88	0.88	-0.5	0.85	0.88	0.88	-0.2	0.94
Region (Ref. Eastern)									
Central	U	0.06	0.25	-54.8	0.00	0.06	0.25	-52.9	0.00
	M	0.06	0.06	-0.3	0.88	0.06	0.07	-0.7	0.66
Western	U	0.47	0.42	10.8	0.00	0.48	0.42	10.8	0.00
	M	0.47	0.48	-0.9	0.72	0.48	0.49	-1.8	0.46

Notes: Ref.=reference; U=unmatched; M=matched; TCM=traditional Chinese medicine; MCH=maternal and child health

Figure S1 Distribution of interaction effects against predicted probability and *z*-statistics using "inteff" command

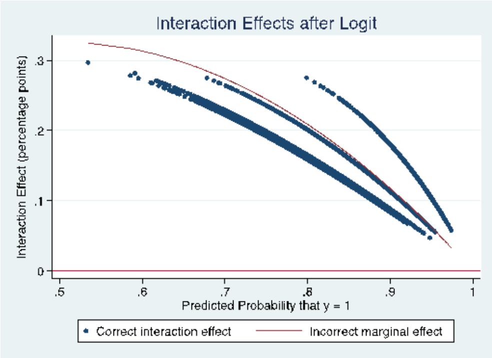

Model 2 (1)

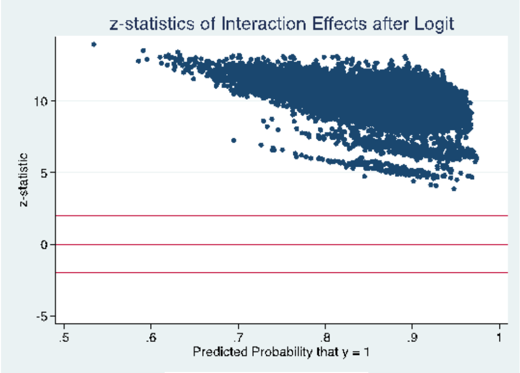

Model 2 (2)

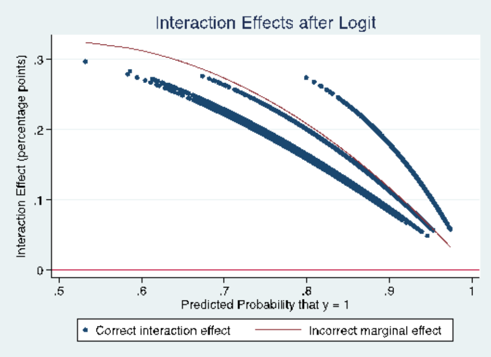

Model 3 (1)

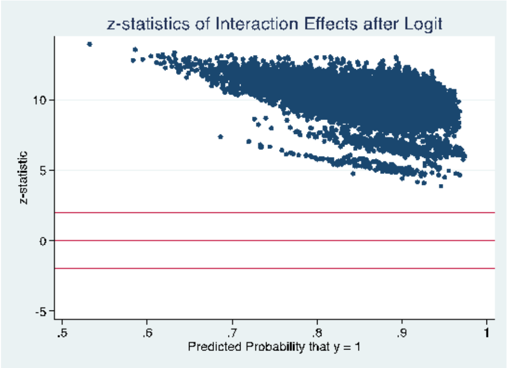

Model 3 (2)

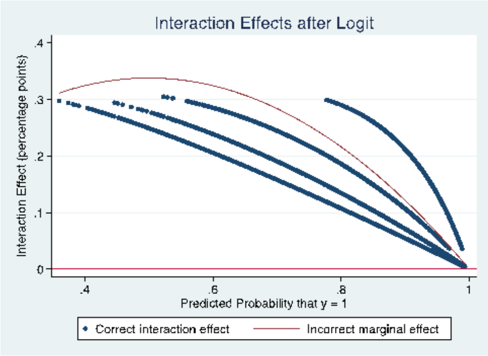

Model 4 (1)

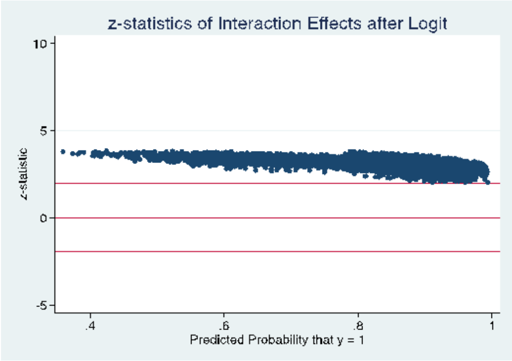

Model 4 (2)
